# The Relation of the White Blood Corpuscles to Suppurative Inflammation

**Published:** 1870-04

**Authors:** A. Henocque

**Affiliations:** No. 6 Sixteenth St.


### THE RELATION OF THE WHITE BLOOD CORPUSCLES TO SUPPURATIVE INFLAMMATION.

By A. HENOCQUE.

Translated from the *Gazette Hebdomidaire* for the Medical Examiner.

Two important communications have been made, one to the Academy of Sciences, by Mr. Robin, in behalf of Mr. Feltz, the other to the Academy of Medicine, by Mr. Vulpian, in behalf of M. Hayem, treating of histological subjects of much interest to the anatomist and pathologist. We propose, therefore, to present to the readers of the *Gazette* a brief *resume* of the results of various experiments which have been made in Germany, England, and France. The question is not one simply of histological and pathological fact; it involves also a theory of inflammation that has been designated "The Theory of Cohnheim." The fact is the passage of the leucocytes, or white blood corpuscles, through the walls of the vessels in the inflamed tissues. The theory is the application of this fact to the history of suppurative inflammation. We shall examine both.

Less fortunate than Cohnheim, and those who have repeated his experiments, the real inventor, A. Waller, had not the good fortune to attract attention to his discovery. Even after the experiments of Cohnheim became known in his own country, it was not England that claimed for him priority, but Hungary. Koloman Balogh, professor at Pesth, first called attention to the works of Waller.

In the year 1846, Waller,* studying the circulation of the blood, in the mesentery of the toad and in the tongue of the frog, discovered the passage of the white and red corpuscles through the walls of the capillary vessels. He concluded, after a prolonged series of experiments, that the white corpuscles

* A. Waller, *Philosophical Magazine*, vol. xxix. Koloman Balogh, *Virchow's Archives*, 45 Bd., p. 19.

could penetrate the vascular walls unchanged; and that the blood possessed the property of closing the openings which had served for their passage.

In another work, published in the same volume, under the title of "Microscopic observations on the perforation of the capillaries by the corpuscles of the blood, and on the origin of mucous and pus globules," Waller established the identity of globules of pus and of mucous with white blood corpuscles. This paper elicited neither criticism nor discussion.

In September, 1867, Cohnheim* published in the *Archives of Virchow*, an extended work, in which he established the fact that pus corpuscles existing in the inflamed cornea are not formed there, but that they have their origin outside of the cornea, and are in fact leucocytes, or white blood corpuscles, which, in virtue of their amiboid movement, pass the walls of the bloodvessels in the cornea.

Cohnheim believed himself the discoverer of this fact. His experiments were made upon the hare and frog, and in the latter, after having colored the white corpuscles by carmine, or blue aniline (which is accomplished by injecting the coloring matter into the lymphatic sacs), he found the white corpuscles containing particles of color in the inflamed cornea.

The same phenomenon was produced upon the cornea of the hare. Cohnheim demonstrates directly, the passage of the white and red corpuscles through the vascular walls of the mesentery of the frog, and describes with care the successive stages of the migration of the leucocytes.

These experiments were soon repeated, and the results have varied upon some points. In order to appreciate them well, it is necessary to separate the observations upon the cornea and the non-vascular tissues, from those made upon structures rich in vessels.

Hoffman and Recklinghausen† observed that in the cornea detached, but preserved by a special arrangement, there were

* Cohnheim. *Archiv. of Patholog., An. et Phi.*, 40 Bd., p. 679.

† Hoffman and Recklinghausen. *Central Blatt*, 1867, No. 31. Hoffman, *Archiv. f. Patho., An. and Phys.*, Bd. 44, p. 204.

produced new elements, having amiboid movements. They, therefore, maintain that in the cornea, pus may be formed, directly at the expense of previously existing elements. These authors do not contradict the facts observed upon the mesentery.

Stricker and Norris † have also concluded from their experiments, that in ceratitis the contractile cells are formed by the multiplication of the elements of the cornea itself. In inflammation of the vascular parts, the very exact experiments of these authors have, on the contrary, fully confirmed the results of Cohnheim.

Prominent among the works recently published is that of Kremiansky, ‡ (January, 1868). As it is less known than the preceding, we shall give a more detailed *resume* of its most important points. The first series of experiments consist of microscopic observations of the inflammatory process in various animals. The author has perfected the manner of operating; and in the mammifera, the white corpuscles being difficult to distinguish, he colors them by injecting in the blood aniline, or better still, cinnabar finely pulverized. A few hours after the injection, the greater part of the white corpuscles contain particles of cinnabar, thus rendering these elements apparent.

Still farther, the author has discovered a means of rendering the phenomenon more rapid, by employing cauterization, especially in the use of the crystals of cantharadine, distributed along the veins. By this means the migration of the leucocytes is accomplished in about ten minutes.

His first observations confirm those of Cohnheim. In fact, Kremiansky has been able to follow, in the mesentery of the frog, the various phases of the process, that is the enlargement of the veins, the retardation of the circulation, the accumulation of the white globules, and, at the border of the blood stream, the stoppage of the white corpuscles against the interior surface of the wall; the appearance of accumulations in the form of spheres, of buttons, of cones, on the exterior of

† Norris et Stricker Vienna, 1869..

‡ Kremiansky. *Weiner Medizinische Wochenschrift,* Nos. 1 to 6, 1868.

the vascular walls; the gradual increase of these accumulations; the complete passage of the white corpuscles out of their vessels, with their successive modifications, into pyriform, fusiform, stellate, pollygonal, and rounded bodies. These globules accumulate around the vessels, then break into the neighboring tissues with change of form, and finally assume the well-known appearance of pus corpuscles.

Still farther, Kremiansky has seen the corpuscles at the same time, pass through the veins and the wall of the lymphatic, by which, in certain cases, it is surrounded. In the capillaries, the white corpuscles commence their migration when the stagnation of the blood is complete; they remain often imbedded in the midst of the walls. The red corpuscles can also pass the capillaries, but they also often remain engaged in the walls of the vessels. In the arteries, the author has never witnessed this migration of the globules.

These results differ from those of Cohnheim in some points of detail. In fact, Kremiansky has seen the passage of the red globules through the veins, as well as through the capillaries. They also undergo this change of relation independently of the white, although Cohnheim believed the red globules only passed the capillaries following the leucocytes. Such are the facts observed in the simple irritation caused by the exposure of the mesentery of the frog to the action of the atmosphere. The phenomena are somewhat different when a local irritation is produced by the application of a crystal of cantharadine to a vein. There are observed, first a narrowing of the vessels at the point of application of the irritant, an acceleration of the inflammatory process, which limits itself at a determined point, the passage of the leucocytes through the wall is much more easily seen, and finally, there is added an important fact; namely, the lymphatic corpuscles which are encountered normally in the conjunctive tissues of the mesentery, change their position, moving towards the irritated part, and accumulating there.

These phenomena may be readily observed in the tongue of the frog. Kremiansky has also observed the same fact in the wing of the bat, and in the inflamed mesentery of the hare and dog; but in the latter, he has not been able to follow directly the ulterior changes of the white corpuscles.

A second series of indirect observations complete the study of the process. After having injected into the veins of the frog, the hare, the dog, the cat, and the fowl, a weak saline solution, containing cinnabar in suspension, he detected the existence of inflammation in various organs, and was able thus to study the position occupied in the tissues, by the leucocytes containing grains of cinnabar. These experiments have not been conducted exclusively upon the non-vascular tissues (cornea cartilages), but have also been made upon the peritonium.

The conclusion of the author may be summed up as follows: The inflammatory products (pus exudations, false membranes, adhesions,) are made up histologically, for the most part, of the white corpuscles which come from the veins and the capillaries, the walls of which they traverse, (*emigration des leucocytes*). On the other hand, the forms contained in the inflammatory products may come from the elements which are contained in the conjunctive tissue of the cornea, or of the mesentery, and which have amiboid movements, and are really the lymphatic cells· Finally, these inflammatory products may be formed in part by a proliferation of the fixed corpucles of the conjunctive tissues, as in the inflamed cartilage, when young cells develop at the expense of pre-existant cells.

In England, Charlton Bastian* published and repeated' the experiments of Cohnhiem. He saw not only the migration of the leucocytes, but that of the red corpuscles, which pass the walls, not by a simple passive phenomenon, but, as he believes, in virtue of an active property. These corpuscles seeming to possess, in themselves, amiboid movements.

In France, the works of Cohnheim have been noticed by a very faithful analysis published in the *Archives de Physiologie Normale et Pathologique*, for March, 1868. Mr. Chalvet, in his inaugural thesis (*Phisiologie Pathologique de l'Inflammation*, 1869), has also noticed these experiments; and more recently the

* C. Bastian. *Medical Times and Gazette*, March 9, 1868.

researches of M. M. Hayem* and Feltz† have been the occasion of the discussion of the subject in the academies. Mr. Hayem has made observations upon the mesentery and lung of the frog, and his descriptions are, in the main, in accordance with the published results of Cohnheim. He has found the epithelial layers and the conjunctive tissues of the mesentery almost perfect. The epithelial covering remains inactive during the process of inflammatiom, and is penetrated by the leucocytes. Following the developement of traumatic pneumonia in the lung of the frog, Mr. Hayem has observed that the inflammatory process presents the same phases as in the mesentery, and that, while the white and red corpuscles are seen to pass through the alveolar walls and partitions, not a single nuclear multiplication in the corpuscles of the inter-capillary spaces is observed. But, on the contrary, a certain number of these elements become transformed into granular bodies, under the eye of the observer.

I will add, that the experiments of M. Hayem have been made, in some sense, publicly, in the laboratory of Mr. Vulpian; so that a number of persons have been able to follow them, and Mr. Vulpian himself has no hesitation in considering the phenomenon of migration as a fact perfectly established, and easy of observation.

We cannot too strongly urge upon histologists the importance of repeating these experiments, and arriving at personal knowledge upon the subject; especially as it can be accomplished at a sacrifice of but little time or patience. For the purpose of verification simply, we would recommend the means employed by Kremiansky, that is the application of crystals of cantharadine upon the mesentery or tongue of the frog. We have been, by this means, able to follow closely the modifications of the circulation of the inflamed parts, and to observe the accumulation of the leucocytes upon the interior surface, and their migration through the wall. In these studies, we confess we sought for the causes of error in Kremiansky's experiments,

* Hayem. *Gazette Medicale*, No. 1, 1870.

† Feltz. *Journal de l'Anatomie et de la Physiologie*, No. 1, 1870.

rather than to verify them, as we were strongly inclined to doubt the correctness of his conclusions. The doctrine has met with strong opposition, not only in its application to the non-vascular tissues, such as the cornea, but the alleged fact itself, of the migration under any circumstances, has been strongly denied. We shall notice only those opponents who attempt to refute the doctrine by experiment.

Koloman Balogh,* in 1868, repeating the experiments upon the mesentery of the frog, did not observe the passage of the white corpuscles through the walls of the vessels. He pointed out certain causes of error, and gave a different interpretation to the appearance of the inflamed mesentery. He also called the attention of the scientific public to the fact that to A. Waller, of England, and not to Cohnheim, is due the credit, if such it be, of priority in the publication of the doctrine.

Mr. Feltz, of Strasbourg, has experimented upon the tongue and mesentery of the frog, and the mesentery of the young mouse; he has also studied the artificial inflammation of the peritonium; tried without success the injection of aniline, and notwithstanding repeated observations, has never seen the white corpuscles pass the vascular walls. Mr. Feltz agrees upon one point with Cohnheim; that is upon the absence of remarkable modifications in the elements of the peritoneal connective tissues. Still farther, he has never seen the extra-vascular elements, which constitute pus, forming themselves of pre-existent elements, whether by direct division or by the multiplication of their nuclei.

This is the state of the question, as to the passage of the leucocytes through the walls of the blood-vessels. Some have seen it, others have not seen it. The negation is an absence of knowledge, and we might rest here. True criticism, however, seeks to look into the details of the experiments, to discover, if possible, the reason for this difference of opinion.

Let us follow Mr. Feltz, step by step, in his argument and observations. One fact strikes us in his descriptions. If we exclude, in the experiments of Cohnheim, the affirmation of the

* Koloman Balogh. *Archives de Physiologie,* No. 1, 1869.

direct passage of the leucocytes, we find a great analogy between the two authors. Indeed, Mr. Feltz has seen the inert layer upon the border of the blood stream, in which the leucocytes slowly circulate, the red corpuscles being in the centre of the current; he has also seen the accumulations of the white corpuscles on the interior surface, their adhesion to this surface, and their change of form; and, finally, he points out also the union of a great number of these elements upon the exterior surface of the wall. That which is wanting is the detection of the white corpuscles in the act of their passage or strangulation in the walls. The presence of these elements upon both sides of the walls involves, perhaps, the theory of their migration.

Mr. Feltz has seen still more. We quote: "After a certain time, there appears at the external border of the vessels, one or two, and even more, little asperities, which, when analyzed with care, are found to be nothing more than little masses of leucocytes, resembling in all particulars those upon the interior of the wall. These little external mamelons, composed of elements perfectly independent of each other, are quite as likely to be produced upon the points corresponding to the interior mamelons as upon other portions of the wall, and I have seen many times, fifteen, twenty, or thirty corpuscles massed into points where there were, upon the interior of the vessels, only a smooth and simple lining of white elements."

Indeed, Mr. Feltz has almost seen the phenomenon complete. It is to be regretted that he has not been more precise in giving exact information as to the volume of the little asperities, and of the little masses, because if they can be resolved into three or four leucocytes, a quantity necessary to form a little mass, it seems to us that they constitute more than a little asperity. The white globules of the frog are about eight to ten-thousandths of a millimetre (one-twenty-five-hundredth to one-thirty-one-hundreth of an inch) in diameter, while the smaller veins are nine-hundredths of a millimetre (one-two-hundred and seventy-fifth of an inch) in diameter. The thickness of the wall is about one-hundredth of a millimetre (one-twenty-fivehundredth of an inch); it follows, then, that the projection formed by three or four leucocytes upon the exterior of the wall, will be at least double the thickness of this wall. But it often happens that the asperities observed upon the exterior of the wall are in reality of the size of a leucocyte, or less, when first discovered. Besides, by observation, one may see masses, as Mr. Feltz has done, transformed into leucocytes assuming the globular form, sometimes two or three in succession, make their appearance. In fact, they follow each other through the vascular walls.

Mr. Feltz has seen, in a very transparent mesentery of a mouse, leucocytes appear at a point transversed by a single vessel, without there having been found a single interior asperity or mamelon in the whole extent of the branch under observation. This is a phenomenon which has been explained by Kremiansky, since according to him, corpuscles analogous to leucocytes, existing normally in the mesentery, are endowed with spontaneous movement, and are seen to converge towards the vessels.

Mr. Feltz, in the second part of his work, endeavors to demonstrate by negative results, that pores permitting the passage of the leucocytes, do not exist in the vascular walls, as these walls do not allow the passage of colored injections, nor of fine particles of aniline. We shall return to this objection, which has a bearing upon the theory of Cohnheim. Thus far we have endeavored to examine carefully the principal fact—the migration of the corpuscles through the vessels, which constitutes the basis of Cohnheim's theory. This theory, however, comprehends the accessory facts and conditions which accompany or explain the passage of the leucocytes, as well as the application of these facts and conditions to the theory of inflammation and of suppuration.

Such are the two new points which we shall proceed to investigate. First, the experimentors who have seen the passage of these corpuscles, are called upon to indicate the mode and anatomical route by which the migration is accomplished. Cohnheim suggests the open mouths of the epithelium of the vessels. Unfortunately this is a point of anatomy still in doubt. The resistance of these walls to the passage of fine particles seems to be inconsistent with the hypothesis of open epithilial mouths. The experiments made with aniline, and with cinnabar, which color more easily, have not, up to the present time, furnished sufficient proof upon this point; it is necessary to wait for further developments before arriving at a conclusion. The three negative experiments of Mr. Feltz are not, in my opinion, of great importance; since, on the one hand, he has not seen the white corpuscles imbued with the coloring matter of aniline—a process of colorization very uncertain,—while, on the other hand, the results of the injection of cinnabar have been very clear, thus proving that the granules penetrate easily into the red corpuscles, and are even deposited in the various tissues. It is so easy to color the white corpuscles with cinnabar, that this fact need not be left in doubt. That which is more difficult to demonstrate is the *manner* by which these granules of cinnabar penetrate the tissues. But is this difficulty as to the *mode* a sufficient reason for believing that the *fact* of the penetration does not exist. No; it is simply a want of knowledge, but cannot negative a single determined fact. As to the red corpuscles, they can only be forced through the walls by pressure of the blood, *a vis a tergo*, for, notwithstanding the opinion of Bastian, and of other experimentors, the amiboid movement of these corpuscles is not yet demonstrated.

There are other conditions which favor the passage of the globules out of the vessels; such as the paralysis of the smooth muscular fibre indicated by the enlargement of the vessels, but the discussion of all these questions would occupy too much time, we must be content to simply suggest them.

Cohnheim endeavored to show that suppuration is always the result of migration, or of the extravasation of the white globules. In his second article,* he insists upon the integrity of the fixed corpuscles of the conjunctive tissues of the cornea and of the mesentery, in his experiments. The pus, therefore, can only be produced from the white corpuscles or leucocytes

* Cohnheim. *Archives of Pathol., Anat. et Phys.*, vol. xlv, p. 333.

which have passed from the blood. He claims that this is the case in both the vascular and non-vascular tissues. The facts which we have cited tend to support this theory, so far as it applies to the vascular tissues. The almost complete integrity of the fixed corpuscles of the conjunctive tissues, is a fact which has been remarked by nearly every experimentor—even Mr. Feltz; but as to the non-vascular tissues, there still remains, as we have seen, much doubt. The reason for these divergences of opinion are easily seen; they depend entirely upon the anatomical question of the texture of the conjunctive tissues.

Since Recklinghausen has shown, in the cornea, in a normal state, the existence of movable corpuscles, which are also found in the mesentery, where they represent cells and embryoplastic nuclei, described by Mr. Robin; other experimentors (Huxley, Kühne, Koelliker) have also seen these elements endowed with amiboides properties or contractions. We have ourselves* followed the changes of form in these elements, in the mesentery of the frog. Finally, Kremiansky attributes to them a certain role in inflammation. The question is complex, and the synthetical study of the connective tissues does not seem likely to simplify it.

The theory of suppuration promulgated by Virchow, and generally adopted, namely, that pus is formed by the proliferation of the conjunctive elements, does not require an exhaustive study or analysis of each of the tissues made up of these elements; now, however, it is necessary to search for the truth by a thorough anatomical study of each of these tissues. We find the evidence of this necessity when we examine the theory of Cohnheim for a definite idea or principle, the demonstration of which is found in the fact of the emigration of the leucocytes.

It seems to us that there is an evident agreement only between the theory and the phenomena observed in the mesentery; that is in the laminated or conjunctive tissues, properly so called, and that we may therefore adopt the following conclusions, analogous to those of Kremiansky:

* Hayem et Henocque. *Archives Gen. de Medicine*, Juin and Juillet, 1866.

*1st.* In suppurative inflammation of the laminated tissue, the act of suppuration is essentially vascular, the leucocytes pass the walls of the vessels, and are the elements called pus corpuscles.

*2d.* The embryoplastic elements, or the mobile corpuscles of the conjunctive tissues, have altogether an accessory part.

*3d.* As to the fixed corpuscles to the laminated fibres, their office is nul. There is no proliferation, in the ordinary sense of the term; there are notable alterations only in case the inflammation passes beyond the limits of suppurative inflammation. To apply these phenomena to tissues of conjunctive substances in general (tissues of the cornea, of bone, etc.), would be, in my opinion, premature. For all these tissues the question is still one for investigation and discussion.

Finally, in looking over the phenomena and theories presented, we see on the one hand, a fact positively affirmed by a good number of observers, and on the other, a theory of which the limits are not yet well defined, and of which many of the elements are incomplete. The fact itself is almost fatal to the two principal theories, which, up to the present time, have been invoked to explain the formation of pus—that is the theory of proliferation, and that of spontaneous generation in the midst of blastema.

It will be readily admitted, that it is important to complete the investigation as to the fact rather than claim its impossibility because it is inconsistent with two theories which are themselves in opposition the one to the other. J.

*No. 6 Sixteenth St., March 7th, 1870.*

---

Something in Regard to Dr. Charles West, the Children's Friend.—The London correspondent of the *Cincinnati Lancet and Observer* called upon the eminent author and teacher, Charles West, M.D., F.R.C.S., and found him exceedingly social and agreeable, and in splendid health, though quite old. He does but little beyond consultation practice. He is now connected with only one hospital, which he founded in Queen's Square, for sick children, and calls it *his child.* Dr. West does not pretend to lecture.—*Medical Record.*

